# Rheumatoid arthritis seropositive for the rheumatoid factor is linked to the protein tyrosine phosphatase nonreceptor 22-620W allele

**DOI:** 10.1186/ar1812

**Published:** 2005-08-25

**Authors:** Philippe Dieudé, Sophie Garnier, Laëtitia Michou, Elisabeth Petit-Teixeira, Elodie Glikmans, Céline Pierlot, Sandra Lasbleiz, Thomas Bardin, Bernard Prum, François Cornélis

**Affiliations:** 1 GenHotel-EA3886, Evry-Genopole, Evry, France; 2 Unité de Génétique Clinique, Fédération de Rhumatologie, Centre Viggo-Petersen, Hôpital Lariboisière, Assistance Publique des Hôpitaux de Paris, Paris, France; 3 Laboratoire Statistique et Génome, Evry-Genopole, Evry, France; 4 Consultation de Génétique Adulte, Centre Hospitalier Sud-Francilien, Evry-Corbeil, France

## Abstract

The protein tyrosine phosphatase nonreceptor type 22 (*PTPN22*) gene encodes for lymphoid tyrosine phosphatase LYP, involved in the negative regulation of early T-cell activation. An association has recently been reported between the *PTPN22-620W *functional allele and rheumatoid factor-positive (RF^+^) rheumatoid arthritis (RA), among other autoimmune diseases. Expected linkage proof for consistency cannot be definitely produced by an affected sib-pair (ASP) analysis. Our aim was therefore to search for linkage evidence with the transmission disequilibrium test.

DNA from the French Caucasian population was available for two samples of 100 families with one RA patient and both parents, and for 88 RA index cases from RA ASP families. Genotyping was carried out by PCR-restriction fragment length polymorphism. The analysis was performed using the transmission disequilibrium test, genotype relative risk and ASP-based analysis.

The transmission disequilibrium test of the *PTPN22*-*620W *allele revealed linkage and association for RF^+ ^RA (61% of transmission, *P *= 0.037). The genotype relative risk showed the risk allele in 34% of RF^+ ^RA patients and in 24% of controls derived from nontransmitted parental chromosomes (*P *= 0.047, odds ratio = 1.69, 95% confidence interval = 1.03–2.78). The ASP investigation showed no enriched risk allele in RA multiplex families, resulting in a lack of power of ASP analysis, explaining the published negative results.

This study is the first to show linkage of *PTPN22 *to RF^+ ^RA, consistent with *PTPN22 *as a new RA gene.

## Introduction

Rheumatoid arthritis (RA), the most common autoimmune disease, is thought to be a complex disease in which a combination of risk alleles from different susceptibility genes predisposes to the development of the disease, following exposure to as yet unknown environmental factors. Several genome scans have suggested multiple RA loci [1-8], and recent case-control association studies have suggested new RA genes [9,10]. However, only *HLA-DRB1 *alleles have been both linked to and associated with RA, fulfilling the criteria for a fully demonstrated genetic factor [11].

A genetic association involving a functional polymorphism of the protein tyrosine phosphatase nonreceptor type 22 (*PTPN22*) gene was reported to be associated with rheumatoid factor-positive (RF^+^) RA, with type 1 diabetes, with systemic lupus erythematosus and with autoimmune thyroid disease [12-19]. The *PTPN22 *gene encodes for the intracellular tyrosine phosphatase LYP, which acts as a negative regulator of early T-cell activation through binding to the Csk protein [20,21].

The *PTPN22 *single nucleotide polymorphism (SNP) (*1858C/T*) (rs 2476601) occurs as a result of an amino acid substitution of arginine for tryptophan at position 620 (R620W), in the P1 proline-rich domain. This domain is involved in the binding to the SH3 domain of Csk. Functional analysis showed that it affects the binding of LYP to Csk, leading to a lack of downregulation of T-cell activation, which is consistent with an increased susceptibility to autoimmunity for the *620W *allele [12,14].

The *PTPN22*-*1858T *allele has been reported to be associated with RF^+ ^RA in several case-control studies [12,18,19,22]. The first study, performed in a white North American population, reported an association between the *PTPN22*-*1858T *allele and RF^+ ^sporadic RA (*P *= 6.6 × 10^-4^). This association was replicated in a different sample with multiplex RA cases (*P *= 5.6 × 10^-8^), the association being restricted to RF^+ ^RA patients [12]. The second study, also performed in a white North American population, compared the frequency of the *PTPN22 *risk allele between the Study of New Onset Rheumatoid Arthritis cohort and the control sample of the previous study [12], observing the association between the *1858T *allele and early RF^+ ^RA. The study also suggested a stronger association for the homozygous genotype *1858T/T *[22]. Three recent case-control studies performed in UK, Spanish and North-American Caucasian populations also found an association between the *PTPN22*-*1858T *allele and RA [18,19,23]. In contrast, the Spanish study observed no dose effect of the suspected allele [18]. The UK study found an increased frequency of the suspected *PTPN22 *allele in the RF^+ ^RA cases and suggested a stronger association for the homozygous genotype [19]. The US study confirmed this association was restricted to RF^+ ^RA and also showed a significantly higher risk for the homozygous genotype [23].

These findings provide strong evidence for the involvement of *PTPN22 *in RF^+ ^RA susceptibility [24]. The linkage proof is so far lacking, however, as the linkage analysis of the North American Rheumatoid Arthritis Consortium RA-affected sib-pairs resource for this *PTPN22 *SNP was inconclusive [12]. The transmission disequilibrium test (TDT), simultaneously investigating linkage and association, is predicted to be more powerful than the affected sib-pair (ASP) analysis in demonstrating linkage for a factor such as *PTPN22 *[25,26]. Three family-based association and linkage studies using TDT analysis were recently reported, providing linkage evidence of *PTPN22 *to type 1 diabetes [15,27,28]. The aim of the present study was to test this *PTPN22 *polymorphism for linkage to RF^+ ^RA in the French Caucasian population, taking advantage of the TDT.

## Patients and methods

### Study design and study population

A TDT linkage study was conducted to investigate the *PTPN22*-*1858C/T *SNP in RA for one Caucasian population. RA patients and family members were recruited through a national media campaign in France, which was followed by the selection of individuals who fulfill the American College of Rheumatology (formerly the American Rheumatism Association) 1987 revised criteria for RA [29], according to the physician in charge of the patient. All clinical data were reviewed by rheumatologists from our team (SL, LM or P Fritz). All individuals provided informed consent and the ethics committee of the Hôpital Bicêtre approved the study.

#### Transmission disequilibrium test RA samples

Inclusion criteria for the two samples of the 100 French Caucasian families investigated here were the participation of one RA patient and both parents, as well as a French Caucasian origin of the family, defined by the four grandparents being French Caucasian. Families with an additional sibling with RA or RA patients who were younger than 18 years old were excluded. RA characteristics of index cases from TDT samples 1 and 2 are summarized in Table 1.

#### Affected sib-pair RA sample

The 88 index RA patients from the French Caucasian ASP families that had been analyzed for a refined genome scan were investigated in this study [1]. Inclusion criteria for the sample of 88 families had been the participation of at least two siblings with RA and of French Caucasian origin, with all four grandparents being of European Caucasian origin. Families with RA patients younger than 18 years old were excluded. Of these 88 families, 81 had two affected siblings, six families had three affected siblings and one family had four affected siblings. Characteristics of the 88 RA index cases investigated in this study are summarized in Table 1. All ASP families had been previously genotyped for two microsatellite markers flanking the *PTPN22 *locus (*D1S418 *and *D1S252*) located at approximately -1 and +3 Mb, respectively, on either side of the *PTPN22 *locus, with heterozygosities of 80% and 81%, respectively [1,30].

### Molecular genotyping methods

Genomic DNA was purified from fresh peripheral blood leukocytes by standard methods [31]. *HLA-DRB1 *typing (Dynal Classic high resolution and Sequence Specific Primers DR low resolution) and subtyping (Dynal Classic high resolution, for HLA-DRB1*01, HLA-DRB1*04, HLA-DRB1*11 and HLA-DRB1*13) were carried out using the PCR sequence-specific primers method (Dynal Biotech, Lake Success, NY, USA).

Genotyping of the *PTPN22*-*1858C/T *SNP was performed by PCR-restriction fragment length polymorphism. The sense and antisense primers were, respectively, 5'-GATAATGTTGCTTCAACGGAATTT-3' and 5'-CCATCCCACACTTTATTTTATACT-3'. The *PTPN22*-*1858C/T *transition at codon 620 eliminates a restriction site for *RsAI *in the *1858T *allele. TDT RA sample 1 and sample 2 genotypes were checked with the PCR-restriction fragment length polymorphism using the *XcmI *enzyme, for which the *1858T *allele creates a restriction site. Each genotype was interpreted independently by two of the investigators (EG and PD).

### Rheumatoid factor status

The RF^+ ^status was provided by the presence of at least one positive RF^+ ^result during the disease course, as determined by latex fixation, by Waaler Rose assay or by laser nephelometry. The RF test was performed at least once for all TDT and ASP RA patients. The anti-cyclic citrullinated peptide status of RA patients was not available.

### Hardy–Weinberg equilibrium check

The Hardy–Weinberg equilibrium of the *PTPN22*-*1858C/T *polymorphism was investigated using a chi-square test with one degree of freedom.

### Analysis

We planned a linkage test of the *PTPN22*-*1858T *allele RA hypothesis, restricted to RF^+ ^RA patients. This hypothesis was first tested using the TDT RA sample 1. In case linkage was observed, or at least suggested, a replication test was planned with the TDT RA sample 2 and a global analysis for all TDT RA families. We also investigated the *PTPN22 *putative genotype in the index ASP RA sample, taking advantage of the linkage data available at the *PTPN22 *locus, as previously described [32].

#### Test for linkage and association in the TDT RA samples

Linkage and association analysis were performed using the TDT [33] and the genotype relative risk (GRR) test [34]. The TDT compares the transmission of the SNP alleles from heterozygous parents to affected offspring, with Mendel's law expectation (50%), using a chi-square test with one degree of freedom. Similar to a case–control study, GRR compares the SNP genotypes distribution in RA cases and in 'controls' (controls are derived from nontransmitted parental chromosomes, for each family), using a chi-square test with the appropriate degree of freedom or the Fisher's exact test. *P *< 0.05 was considered significant.

#### Linkage-based test in the ASP RA sample [32]

Genetic factors are expected to be concentrated in families with multiples RA cases, such as ASP families. Within index RA cases, those sharing identical by descent chromosomes at the *PTPN22 *locus with their RA affected sib could be expected to concentrate further *PTPN22 *RA genetic factors. The putative *PTPN22 *genotypes were compared between the ASP RA index cases, the TDT RA cases and the controls from the TDT RA samples (controls are derived from nontransmitted parental chromosomes). For the linkage-based association test, the RF^+ ^index cases that shared at least one allele identical by descent with their RF^+ ^RA sib (IBD1 or IBD2) were used, taking advantage of the linkage data available at the *PTPN22 *locus [32].

#### Stratified linkage analysis based on the PTPN22-1858C/T genotypes

We conducted a linkage analysis using Allegro 1.1 software [35], taking into account the *PTPN22*-*1858C/T *genotypes, to select the subgroup of families with an index carrying the putative genotypes.

### Power calculation

Assuming a *PTPN22*-*1858T *allele association similar to that of the North American population (14.8% allele frequency in RF^+ ^RA cases and 8.7% in controls) [12], association analysis of our 100 TDT families (TDT RA sample 1) provides a 95% power to show a suggestion for association and a 53% power to reach statistical significance (*P *< 0.05). Our sample of 200 TDT families provides a 79% power for significance.

## Results

### Hardy–Weinberg equilibrium check

The *PTPN22*-*1858C/T *polymorphism was in Hardy-Weinberg equilibrium in the control samples investigated.

### Test for linkage and association in the TDT RA samples

#### TDT RA sample 1

The *PTPN22-1858T *allele was more frequent in the RF^+ ^RA cases than in the controls: 20% versus 11% (*P *= 0.022, odds ratio [OR] = 2.05, 95% confidence interval [CI] = 1.1–3.8). The allele frequency observed in rheumatoid factor-negative (RF^-^) RA patients was 16%, compared with 10.5% in the resulting controls (*P *= 0.52). Significant linkage to RF^+ ^RA was observed with an excess of transmission of the *1858T *allele from heterozygous parents to RA cases (66% versus 50%, *n *= 47, *P *= 0.029) (Table 2). The GRR analysis revealed a statistically significant increase in the frequency of genotypes carrying the *PTPN22-1858T *allele (*1858C/T *+ *1858T/T*) in RF^+ ^RA cases (35%) compared with controls (21%) (Table 3).

#### TDT RA sample 2

We observed an excess of transmission of the *PTPN22-1858T *allele to RF^+ ^RA patients that was not significant (56%, *P *= 0.45) (Table 2). The GRR analysis showed a nonsignificant increase of the genotypes carrying the *1858T *allele in RF^+ ^RA patients compared with controls (Table 3).

#### Combined analysis of TDT samples

No statistically significant difference was observed between samples 1 and 2, allowing pooling for combined analysis. *PTPN22 *linkage to RF^+ ^RA was significant (T-allele transmission, 61%; *n *= 90, *P *= 0.037). By contrast, transmission to RF^- ^RA followed Mendel's law exactly (50%) (Table 2). The *PTPN22-1858T *allele frequency was significantly increased in RF^+ ^RA compared with controls (19% versus 13%, *P *= 0.029, OR = 1.62, 95% CI = 1.05–2.50). The GRR analysis showed a significant increase of *PTPN22 *genotypes carrying the *PTPN22-1858T *allele in RF^+ ^RA patients compared with controls (34% versus 24%, *P *= 0.047, OR = 1.69, 95% CI = 1.03–2.78). In the RF^- ^RA patients, genotype frequencies were identical to those of controls, in keeping with the 50% transmission (Table 3).

No correlation between the *HLA-DRB1 *shared epitope status (DRB1*0101, DRB1*0102, DRB1*0401, DRB1*0404, DRB1*0405, DRB1*0408, DRB1*1001) and the *PTPN22 *genotypes in RF^+ ^RA index cases was observed (Table 4). Apart from the RF status, no specific clinical features (erosive disease, age at disease onset) were found to be associated with the *PTPN22-1858T/C *or *PTPN22-1858T/T *genotypes (data not shown).

### Linkage-based test in the RA multiplex ASP sample

The frequency of the *PTPN22-1858T *allele was similar in the RF^+ ^ASP RA cases compared with the RF^+ ^TDT RA cases (18% versus 19%). The linkage-based subgroup (IBD1 or IBD2) of RF^+ ^RA index cases with concordant RF^+ ^RA sibs showed no increase in the frequency of the suspected allele, compared with RF^+ ^TDT RA cases. The GRR analysis was consistent with those findings, the *PTPN22-1858C/T *or *PTPN22-1858T/T *genotype frequency being equal between RF^+ ^RA ASP index cases and RF^+ ^RA TDT cases (Table 5).

### Stratified linkage analysis at the PTPN22 locus based on the 1858C/T genotypes

Previous linkage analysis of these ASP families had shown no linkage at the *PTPN22 *locus (*P *= 0.74) [1]. Stratified linkage analysis in RF^+^-concordant ASP, even after selection of the families with index cases carrying the *PTPN22-1858C/T *or *PTPN22-1858T/T *genotypes, still showed no linkage evidence at the *PTPN22 *locus (*P *= 0.69).

## Discussion

We searched for the *PTPN22-1858T *allele linkage to RF^+ ^RA using the TDT, which simultaneously tests linkage and association, avoiding the major drawback of inevitable imperfect matching between cases and controls. Here, we provide linkage evidence for RF^+ ^RA to the *PTPN22-1858T *allele. We also observed association with the *PTPN22-1858C/T *or *PTPN22-1858T/T *genotypes and we report for the first time an estimation of the association in the French Caucasian population for RF^+ ^RA (34% versus 24%, *P *= 0.047, OR = 1.69, 95% CI = 1.03–2.78). In ASP RF^+ ^RA index cases, the *1858C/T *or *1858T/T *genotype has a similar frequency as the TDT RF^+ ^RA index cases. The association appears to be independent from the *HLA-DRB1 *shared epitope.

Our findings therefore provide linkage evidence in support of *PTPN22 *as a new RF^+ ^RA genetic factor, concurring with previously reported case–control studies [12,18,19,22,23]. We extend this observation to the French Caucasian population, in which the magnitude of the association is similar.

The linkage evidence provided by this study remains statistically modest. Further linkage studies are needed to definitively establish linkage of the *PTPN22-1858T *allele to RF^+ ^RA. For the observed transmission disequilibrium of 61%, a TDT sample size of 232 families would be required to obtain, with 80% power, an independent replication of the linkage evidence reported here.

Genome scans are popular as they do not require any *a priori *hypothesis to detect disease loci. They are clearly unable to detect all disease loci, however, especially factors such as *PTPN22*. The increased power of the TDT over the ASP analysis for such factors was predicted long ago [26]. Our observation of the absence of a major increased frequency of the risk allele in multiplex ASP families when compared with sporadic cases, as reported by Begovich and colleagues [12], allows us to estimate the excess of allele sharing expected in the ASP analysis over the Mendel expectation of 50%. Using the estimation of the divergence from Mendel's law obtained from this study (61% transmission from heterozygous parents to RF^+ ^RA patients, instead of 50%) and the genotype frequencies observed, the allele sharing expected is 52% for all families, 53% for the subgroup of RF^+^-concordant families and 56% for the small subgroup of RF^+^-concordant families with the *1858C/T *or *1858T/T *index case.

A huge sample size would therefore be required to demonstrate a significant excess of allele sharing over Mendel's law. In that regard, the *PTPN22 *situation is similar to that of the insulin gene in type 1 diabetes, for which the discrepancy between numerous association reports and the absence of linkage in ASP analysis was resolved using a TDT-like analysis [36]. This explains the complete absence of linkage evidence that we observed in our ASP analysis, in keeping with the absence of clear ASP linkage reported by Begovich and colleagues [12]. Further analysis using sophisticated software such as GIST might help clarify this point [37].

As indicated by Begovich and colleagues, the chromosome 1 linkage suggestion observed in the ASP analysis of the North American Rheumatoid Arthritis Consortium genome scan is not explained by the findings of the *PTPN22 *association [12]. New RA genes detected by such linkage suggestions, which could be expected to be stronger RA factors, remain to be discovered. Hence the major interest in genome scan persists, despite the lack of power for some RA genes, such as *PTPN22*.

Interestingly, transmission of the *1858T *allele to RF^- ^RA cases precisely followed Mendel's law, with genotype frequencies identical to controls, strengthening the evidence that the *PTPN22-620W *role is restricted to RF^+ ^RA [12,19,22,23]. *PTPN22 *is probably the first example of a fully confirmed RA gene involved specifically in a precise aspect of RA clinical heterogeneity (RF^+ ^RA). The absence of correlation between *PTPN22 *and *HLA-DRB1 *genotypes suggests that both RA genes could be involved in distinct gene combinations predisposing to RA, providing the first example of a clear genetic heterogeneity in RA.

Because the association is relatively modest, no genetic testing would be clinically indicated. Instead, the clinical relevance of the finding is likely to come through the better understanding of RA pathophysiology. It may lead to new therapeutic targets, aiming at the cause of RA, possibly shared by other autoimmune diseases.

Interestingly, all autoimmune diseases reported to be associated with the *PTPN22-1858T *allele are characterized by the production of autoantibodies [12-14,16,22], suggesting that the *620W *variant of LYP could be implicated not only in T-cell activity regulation, but also in B-cell autoreactivity [24]. It will consequently be of major interest to test further for association of the *PTPN22-1858T *allele in RA families with a clustering of multiple autoimmune diseases to measure precisely this association with each disease [38-41].

## Conclusion

Our findings provide linkage evidence for the involvement of the *PTPN22-1858T *allele in RF^+ ^RA genetic susceptibility, in the French Caucasian population, independent of *HLA-DRB1*. This is in keeping with the proposal of *PTPN22 *as a new RA susceptibility gene.

## Abbreviations

ASP = affected sib-pair; GRR = genotype relative risk; PCR = polymerase chain reaction; RA = rheumatoid arthritis; RF = rheumatoid factor; SNP = single nucleotide polymorphism; TDT = transmission disequilibrium test.

## Competing interests

The author(s) declare that they have no competing interests.

## Authors' contributions

PD and SG carried out the molecular genetic studies. LM, EP-T, EG, CP, SL and FC performed acquisition of the data. PD, SG, LM, CP, BP and FC analyzed and interpreted the data. LM, SL and TB made a substantial contribution to the acquisition of clinical data and the follow-up of the patients. All authors read and approved the final manuscript.

## Figures and Tables

**Table 1:** Characteristics of rheumatoid arthritis (RA) index cases from the investigated samples

	TDT RA sample 1 (*n *= 100)	TDT RA sample 2 (*n *= 100)	ASP RA sample (*n *= 88)
Female (%)	87	90	84
Mean age (± standard deviation) at disease onset (years)	32 (± 10)	31 (± 6)	40 (± 14)
Mean (± standard deviation) disease duration (years)	18 (± 7)	16 (± 8)	23 (± 10)
RA patients with bone erosions (%)	90	79	80
RA patients seropositive for rheumatoid factor (%)	81	76	84
RA patients carrying at least one *HLA-DRB1 *shared epitope allele^a ^(%)	78	80	77

TDT, transmission disequilibrium test; ASP, affected sib-pair

^a^DRB1*0101, DRB1*0102, DRB1*0401, DRB1*0404, DRB1*0405, DRB1*0408, DRB1*1001.

**Table 2:** Linkage analysis of the *PTPN22-1858T *allele to rheumatoid factor-seropositive (RF^+^) rheumatoid arthritis (RA) using the transmission disequilibrium test (TDT)

Families	Transmission [% (*n*)]	*P*
TDT RA sample 1		
TDT RA index cases (*n *= 100)	66 (55)	0.022
TDT RA index cases RF^+ ^(*n *= 81)	66 (47)	0.029
TDT RA index cases RF^- ^(*n *= 19)	63 (8)	0.48
TDT RA sample 2		
TDT RA index cases (*n *= 100)	53 (57)	0.69
TDT RA index cases RF^+ ^(*n *= 76)	56 (43)	0.45
TDT RA index cases RF^- ^(*n *= 24)	43 (14)	0.59
All TDT RA families		
All TDT RA index cases (*n *= 200)	59 (112)	0.059
All TDT RA index cases RF^+ ^(*n *= 157)	61 (90)	0.037
All TDT RA index cases RF^- ^(*n *= 43)	50 (22)	1

RF^-^, seronegative for rheumatoid factor. Transmission, percentage of heterozygous *1858C/T *parents transmitting the *1858T *allele. The plan was to test the hypothesis for RF^+ ^RA; analysis for all RA and RF^- ^RA were provided for the discussion.

**Table 3:** Association of *PTPN22 *genotypes carrying the *1858T *allele and rheumatoid factor-seropositive (RF^+^) rheumatoid arthritis (RA)

	*PTPN22 *genotypes [% (*n*)]				*P*^a^	Odds ratio (95% confidence interval)
			
	*C/C*	*C/T*	*T/T*	*C/T *or *T/T*		
TDT RA sample 1						
All TDT RA index cases (*n *= 100)	65 (65)	31 (31)	4 (4)	35 (35)		
Controls^b ^(*n *= 100)	79 (79)	20 (20)	1 (1)	21 (21)	0.029	2.05 (1.07–3.81)
TDT RA index cases RF^+ ^(*n *= 81)	64 (52)	31 (25)	5 (4)	36 (29)		
Controls^b ^(*n *= 81)	79 (64)	20 (16)	1 (1)	21 (17)	0.038	2.1 (1.04–4.24)
TDT RA index cases RF^- ^(*n *= 19)	68 (13)	32 (6)	0	32 (6)		
Controls^b ^(*n *= 19)	79 (15)	21 (4)	0	21 (4)	0.71	
TDT RA sample 2						
All TDT RA index cases (*n *= 100)	69 (69)	27 (27)	4 (4)	31 (31)	0.76	
Controls^b ^(*n *= 100)	71 (71)	26 (26)	3 (3)	29 (29)		
TDT RA index cases RF^+ ^(*n *= 76)	68 (52)	28 (21)	4 (3)	32 (24)		
Controls^b ^(*n *= 76)	74 (56)	24 (18)	2 (2)	26 (20)	0.47	
TDT RA index cases RF^- ^(*n *= 24)	71 (17)	25 (6)	4 (1)	29 (7)		
Controls^b ^(*n *= 24)	63 (15)	33 (8)	4 (1)	37 (9)	0.76	
All TDT RA families						
All TDT RA index cases (*n *= 200)	67 (134)	29 (58)	4 (8)	33 (66)		
Controls^b ^(*n *= 200)	75 (150)	23 (46)	2 (4)	25 (50)	0.078	
TDT RA index cases RF^+ ^(*n *= 157)	66 (104)	29 (46)	5 (7)	34 (53)		
Controls^b ^(*n *= 157)	76 (120)	22 (34)	2 (3)	24 (37)	0.047	1.69 (1.03–2.78)
TDT RA index cases RF^- ^(*n *= 43)	70 (30)	28 (12)	2 (1)	30 (13)		
Controls^b ^(*n *= 43)	70 (30)	28 (12)	2 (1)	30 (13)	1	

RF^-^, seronegative for rheumatoid factor.

^a^Following data previously reported in RA and because of the infrequency of the *PTPN22-1858T/T *genotype, it was combined with the *1858C/T *genotype for the analysis.

^b^Controls derived from nontransmitted parental chromosomes.

**Table 4:** *PTPN22-1858 C/T *genotypes distribution according to the *HLA-DRB1 *shared epitope (SE)

	*PTPN22 C/T or T/T*	*PTPN22 C/C*	*P*
TDT RA sample 1			0.88
HLA-DRB1*SE/SE	10	16	
HLA-DRB1*SE/X	18	34	
HLA-DRB1*X/X	7	15	
TDT RA sample 2			0.16
HLA-DRB1*SE/SE	13	17	
HLA-DRB1*SE/X	14	35	
HLA-DRB1*X/X	4	17	
All TDT RA families			0.25
HLA-DRB1*SE/SE	23	33	
HLA-DRB1*SE/X	32	69	
HLA-DRB1*X/X	11	32	

HLA-DRB1*SE/SE, two shared epitopes; HLA-DRB1*SE/X, one shared epitope; HLA-DRB1*X/X, zero shared epitope.

**Table 5:** *PTPN22-1858C/T *genotypes frequencies in the affected sib-pair (ASP) rheumatoid arthritis (RA) sample

	*PTPN22-1858C/T *genotype frequencies [% (*n*)]				*P*^a^
		
	*C/C*	*C/T*	*T/T*	*C/T *or *T/T*	
ASP RA index cases RF^+ ^(*n *= 74) versus controls^b ^(*n *= 200)	66 (49)	31 (23)	3 (2)	34 (25)	0.15
ASP RA index cases RF^+ ^and IBD1 or IBD2 at the *PTPN22 *locus with their RF^+^-concordant RA sib (*n *= 42) versus controls^b ^(*n *= 200)	71 (30)	26 (11)	3 (1)	29 (12)	0.63
All ASP RA index cases (*n *= 88) versus controls^b ^(*n *= 200)	69 (61)	29 (25)	2 (2)	31 (27)	0.32

RF^+^, seropositive for rheumatoid factor; IBD1 or IBD2, index sharing 1 or 2 identical by descent allele with the RA sib.

^a^Following data previously reported in RA and because of the infrequency of the *PTPN22*-*1858T/T *genotype, it was combined with the *1858C/T *genotype for the analysis.

^b^Controls derived from all non transmitted parental chromosomes.
